# Modulations in Chlorophyll *a* Fluorescence Based on Intensity and Spectral Variations of Light

**DOI:** 10.3390/ijms23105599

**Published:** 2022-05-17

**Authors:** Edappayil Janeeshma, Riya Johnson, M. S. Amritha, Louis Noble, K. P. Raj Aswathi, Arkadiusz Telesiński, Hazem M. Kalaji, Alicja Auriga, Jos T. Puthur

**Affiliations:** 1 Plant Physiology and Biochemistry Division, Department of Botany, University of Calicut, C.U. Campus, Tenhipalam 673635, Kerala, India; janeeshma_dob@uoc.ac.in (E.J.); riyajohnson@uoc.ac.in (R.J.); amrithams@uoc.ac.in (M.S.A.); noble_louis@uoc.ac.in (L.N.); aswathirajkp1@gmail.com (K.P.R.A.); 2 Department of Bioengineering, West Pomeranian University of Technology in Szczecin, 71-434 Szczecin, Poland; arkadiusz.telesinski@zut.edu.pl; 3 Institute of Technology and Life Sciences—National Research Institute, Falenty, 05-090 Raszyn, Poland; hazem@kalaji.pl; 4 Department of Plant Physiology, Institute of Biology, Warsaw University of Life Sciences SGGW, 02-776 Warsaw, Poland; 5 Department of Animal Anatomy and Zoology, Faculty of Biotechnology and Animal Husbandry, West Pomeranian University in Szczecin, 71-270 Szczecin, Poland; alicja.auriga@zut.edu.pl

**Keywords:** blue light, chlorophyll *a* fluorescence, F_V_/F_M_, high light, low light, photosynthesis, red light, UV-B exposure

## Abstract

Photosynthetic efficiency is significantly affected by both qualitative and quantitative changes during light exposure. The properties of light have a profound effect on electron transport and energy absorption in photochemical reactions. In addition, fluctuations in light intensity and variations in the spectrum can lead to a decrease in photosystem II efficiency. These features necessitate the use of a simple and suitable tool called chlorophyll *a* fluorescence to study photosynthetic reactions as a function of the aforementioned variables. This research implies that chlorophyll *a* fluorescence data can be used to determine precise light conditions that help photoautotrophic organisms optimally function.

## 1. Introduction

Photosynthesis depends on light sources, and the intensity and spectral distributions of light exposure greatly influence photosynthesis rate [1]. The number of photons or the intensity of light is directly proportional to the quantity of light. Based on the evaluation of photosynthesis rate, growth parameters, carbohydrate biosynthesis, and leaf orientation, the optimal light conditions for the maximum growth of plants was fixed in the range of 400 and 500 mol m^2^ s^1^. Photosynthesis was reduced when the plant was exposed to low light (100–300 mol m^2^ s^−1^) and high light (700 mol m^2^ s^1^) [2]. Plant species, organism habitat, water availability, CO_2_ content, and other abiotic elements can influence the ideal light intensity reaching the plant. The spectrum characteristics of light, such as the quality of light, play an important role in the rate of photosynthesis. As the dominant portion of the electromagnetic spectrum used by plants for photosynthesis falls in the spectral range 400–700 nm, it is commonly referred to as photosynthetically active radiation (PAR). Plant photosynthesis is generally most efficient in the 425–456 nm and 600–700 nm PAR ranges [3]. However, variations in light availability due to differences in natural habitats create deviations from the PAR range, which has an impact on plant photosynthetic activity. As a result, autotrophic photosynthetic reactions must be screened according to the quality and quantity of light received by the plants [4].

**Figure 1:**
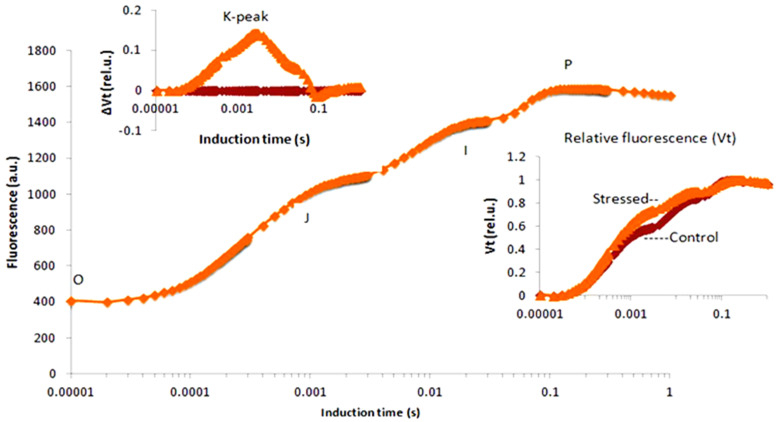
Chlorophyll *a* fluorescence parameters related to the OJIP curve.

Precision and non-invasive procedures are required to assess photosynthetic reactions, with chlorophyll *a* fluorescence providing superior input over other techniques. The high light tolerance of plants was studied using chlorophyll *a* fluorescence parameters, and it was discovered that the OJIP parameters are highly related to PSII activity [5]. Thomas et al. also analysed the UV-B tolerance level of plants based on chlorophyll *a* fluorescence and interpreted the OJIP parameters in response to UV-B irradiation [6]. For revealing dynamism in the rate of photosynthesis, varying parameters such as F_O_ (basal fluorescence/First step of chl *a* fluorescence kinetics), F_J_ (rate of fluorescence at 2 ms after exposure to the actinic light pulse), F_I_ (rate of fluorescence at 30 ms after exposure to the actinic light pulse), F_M_ (maximal fluorescence level/final phase of chl *a* fluorescence kinetics), K-peak (rate of fluorescence at 0.3 ms after exposure to the actinic light pulse), Area (area above the fluorescence transient curve), and t_FM_ (time needed to attain F_M_) were selected, and these parameters are represented in Figure 1.

Parameters such as F_V_ (maximal variable fluorescence (F_M_-F_O_)), F_V_/F_M_ (maximum quantum yield of PSII photochemistry), F_V_/F_O_ (maximum efficiency of the oxygen-evolving complex), S_M_ (indicates number of electron carriers), N (rate of reduction/oxidation of Q_A_), SFI _ABS_ (related to PS II structure and functioning), PI _ABS_ (performance index of PS II on absorption basis), PI _TOTAL_ (performance index of electron flux to the final PS I electron acceptors), the coefficient of photochemical (qP) and non-photochemical (NPQ) quenching, the efficiency of excitation capture by open PSII centre (FV′/FM′), RC/CS_M_ (density of active PS II reaction centres per cross-section), and DF_ABS_ (driving force index related to PSII absorption basis) were selected to examine the role of light characteristics in photosynthesis. The yield parameters φPo (maximum quantum yield of primary PSII photochemistry), φ (Do) (quantum yield of energy dissipation), φ(Eo) (quantum yield (at t = 0) for electron transport from QA^−^ to plastoquinone), ψo (probability that a trapped exciton will transfer an electron into the electron transport chain beyond QA), and the quantum efficiency of PSII (ΦPSII) were analysed to find out the light-stress-induced photosynthetic changes [7].

Specific energy fluxes such as ABS/RC (absorption flux per RC related to its apparent antenna size), TR/RC (trapping flux leading to QA reduction), ET/RC (electron transport flux from QA^−^ to plastoquinone per RC), and DI/RC (dissipated energy flux per RC) were also analysed in this review. The phenomenological energy fluxes, ABS/CS (absorption of energy per excited cross-section), TRo/CS (excitation energy flux trapped by PSII of excited cross-section), ETo/CSm (electron flux transported by PSII of excited cross-section), and DIo/CSm (heat dissipation of excitation energy by PSII of excited cross-section), were scrutinized for analysing photosynthetic modulations [7]. This study is a comprehensive approach to analysing chlorophyll *a* fluorescence responses related to light characteristics and improving the knowledge in this specific area to encourage future research in the same.

## 2. General Properties of Light

Light is the primary energy source for all living organisms. Plants need light for carrying out photosynthesis. Thus, light plays a crucial role in sustaining life [8].

**Figure 2:**
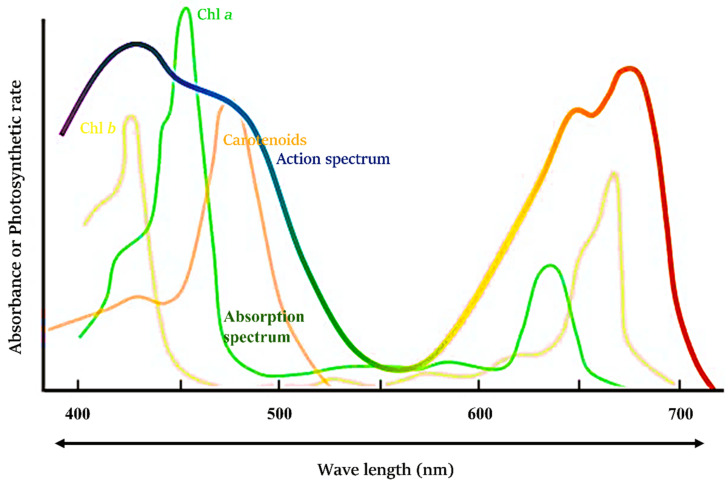
Absorption and action spectra of light in plants.

Considering its particle nature, light is a photon, and light energy is delivered in discrete packets called quanta, which are categorized according to their frequencies. Thus, light possesses both particle and wave properties. Sunlight is similar to a shower of photons with varying frequencies. It is divided into the electromagnetic spectrum, of which a small portion of light between the shorter wavelength UV region (400 nm) and the relatively longer wavelength IR region (750 nm) is visible to humans [9]. Sunlight is the major source of light, and there is a decline in the amount of sunlight striking the earth’s surface and being absorbed by plants. The absorption spectrum is the range of wavelengths of light absorbed by a molecule or substance. Red and blue light are absorbed by plants; however, they reflect green light, giving the plants their green colour. Each photosynthetic pigment has a specific absorption spectrum that is related to the efficiency of its specific wavelength absorption, whereas the action spectrum explains the relationship between the photosynthesis rate and the wavelength absorbed (Figure 2) [10]. Together, the action spectra and absorption spectra of photosynthetic pigments adequately represent the role of light in plant photosynthesis.

## 3. Light and Photosynthesis

### 3.1. Light as the Source of Energy in Photosynthesis

Photosynthesis is the process of converting solar (sun light) energy to chemical energy, in which CO_2_ is fixed as a carbohydrate and energy is stored in chemical bonds [11]. The major roles of light in photosynthesis are (i) oxidation of H_2_O in the oxygen-evolving complex (OEC) and (ii) excitation of photosystem I and II reaction centre chlorophylls [12,13]. The discovery of the well-known Z-scheme paved the way for a greater understanding of the significance of light energy. In photosystem II, water oxidation occurs mainly in the Mn_4_Ca metal cluster at the OEC, which liberates molecular oxygen electrons, protons, and electrons with the help of light energy. In addition, recent studies based on quantum mechanics reveal a clear-cut idea on different oxidation states and the electronic and structural properties of the catalytic cycle involved in them [12]. The photosystem’s light-harvesting complex (LHC) contains antenna proteins, such as carotenoids and chlorophyll *a* and *b* molecules [14,15]. The P680 reaction centre in PSII preferentially absorbs light from 680 nm red light, which is sufficient for oxidising water molecules and also for P680 excitation, but the P700 in PSI absorbs red light far over 680 nm [16]. The combination of the reaction to light and dark together with ETC, followed by ATP generation, continues the energy flow and carbohydrate assimilation [17,18,19]. These had a major role in increasing our understanding of the fundamental mechanisms of photosynthesis, the responses of plants to environmental change, genetic variation, and ecological diversity.

### 3.2. The Mechanisms of Light Interactions in Photosynthetic Efficiency of Plants

Plants have pigment–protein complexes, such as chlorophyll, PSII, and PSI, as well as the light-harvesting complexes (LHCs) that are linked with each reaction centre for the effective absorption and efficient utilization of energy. The energy absorbed in the form of light by the pigments will be used to drive photosynthesis and/or be re-emitted as heat or light (fluorescence). The photochemical reactions and fluorescence do not proceed in a sequential manner, but rather compete. As a consequence, the results of chlorophyll fluorescence emission provide us with information regarding photochemistry, quantum efficiency, and heat dissipation, all of which must be fully comprehended if efforts to improve photosynthesis and plant productivity are to be made [20].

**Figure 3:**
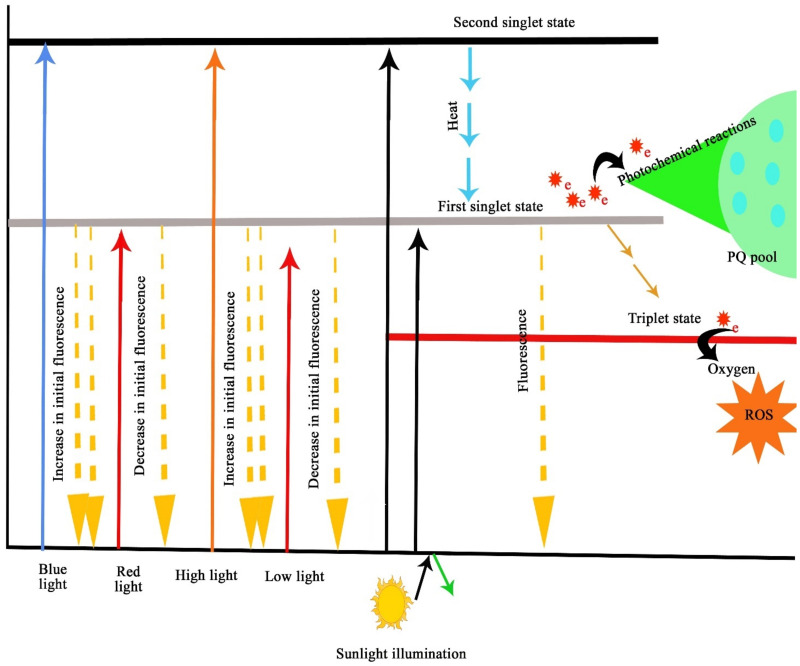
Mechanism of chlorophyll fluorescence in relation to light characteristics.

Light or photons excite the electrons in a chromophore, and the light’s photosynthetic action is wavelength-specific. The excitation induces the delocalization of double bonds in the chlorophyll *a* molecules, and the electrons are shifted to the higher orbit, where this electron shift is dependent on the side chains of the chromophores [21]. Generally, chlorophyll molecules have two excitation states, the first and the second singlet state (Figure 3). A chlorophyll molecule absorbing red light will be shifted to the first singlet state, and the chlorophyll molecule absorbing blue light will be shifted to the second singlet state, which is a highly unstable state. The chlorophyll molecule in the second singlet state reaches the first singlet state (stable as compared to the second singlet state) by energy dissipation [22]. From this state, the chlorophyll molecule transfers the energy to photochemical reactions or emits the excess energy as fluorescence. The excited chlorophyll molecules emit the energy as heat and attain triplet state, where this electron does not participate in photosynthesis but transfers the energy to oxygen and generates reactive oxygen species (ROS).

High light exposure alters the normal light reactions, which increases the number of oxidized PSI that aid in photo-inhibition and the migration of LHC towards PSII for effective quenching of the excess absorbed energy. With this, the fluorescence emission increases, and it is reflected in the initial phase of the chlorophyll *a* fluorescence transient [23]. However, under low light exposure, the OJ phase exhibits a reduction, and the initial fluorescence emission is reduced [24]. On high light exposure, the fluorescence emission at the IP phase of the OJIP curve is low compared to control; at the same time, fluorescence emission at F_M_ or the IP phase of the OJIP curve is high for low-light-exposed plants. Variations in the IP phase indicate an insufficient distribution of light energy to PSI [24].

Visible light with a wavelength of 400 to 700 nm is commonly referred to as photosynthetically active radiation (PAR). When applied as a single wave band, light with a wavelength shorter than 400 nm or longer than 700 nm is regarded as insignificant for photosynthesis due to its low quantum yield of CO_2_ assimilation. Light in the red area (600–700 nm) results in the best quantum yield of CO_2_ uptake by plants. The quantum yield of light in the green range (500–600 nm) is slightly higher than that of light in the blue area (400–500 nm) [25]. Green light has a low absorbance, which contributes to its low quantum yield of CO_2_ by absorbing the least amount of light [26]. Photon absorption by chloroplasts at the adaxial surface may result in heat dissipation of surplus excitation energy, which would enable minimal excitation energy reading by the chloroplasts [27,28]. As a result, blue and red photons would be used inefficiently and would be more likely to be lost as heat than green photons.

The re-emitted light from PSII during the transition from excited to non-excited states is known as chlorophyll fluorescence. Any ambient light can interfere with fluorescence measurements; hence, many early systems had to be used in complete darkness with carefully regulated light settings. The advent of modulating systems, in which the light utilized to cause fluorescence (the measuring beam) is applied at a given frequency (modulated) and the detector is configured to measure at the same frequency, eventually solved this problem. As a result, the detector will only detect fluorescence caused by excitation by the measurement beam, with no interference from ambient light. The obvious benefit is that measurements may be taken without the room darkening [29]. Flash experiment is essential to understand the oxidation and reduction reactions between PSII and Q_A_ [22].

Photosynthetic efficiency, notably in photosystem II (PSII), the water–plastoquinone oxidoreductase, may be quickly measured using chlorophyll *a* fluorescence, which is very sensitive, non-destructive, and dependable. Several studies have found a negative correlation between PSII activity and the fast (up to 2 s) chlorophyll fluorescence rise, as well as the empirical use of fluorescence-rise kinetics in understanding photosynthetic reactions, notably in PSII. The Kautsky effect, named after Hans Kautsky, the discoverer of the phenomenon indicating the existence of fluctuating fluorescence, occurs when dark-adapted photosynthetic samples are exposed to light. In less than 1 s, the chlorophyll fluorescence intensity climbs from a low point (the O level) to a high point (the P-level) via two intermediate phases, labelled J and I. Then, there’s a drop to a lower semi-steady-state level, the S level, which takes roughly a minute to reach [22].

Liu and Iersel found that the light spectrum and photosynthetic photon flux density (PPFD) have an interacting influence on leaf photosynthesis. QYinc (quantum yield of CO_2_ assimilation on the basis of incoming light) was lowest under green light and highest under red light when the PPFD was low. Low absorbance was the cause of the poor QYinc under green light and low PPFD. Green and red light, on the other hand, had similar QYinc at high PPFD, which was higher than that of blue light. Chlorophyll’s greater absorption of blue light results in a considerable light gradient from the top to the bottom of the leaves. Non-photochemical quenching is upregulated at the adaxial side of a leaf due to a large amount of excitation energy, whereas chloroplasts near the bottom of a leaf receive minimal excitation energy under blue light. The light-dependent reactions are a result of the more uniform distribution of green light absorption within leaves, further leading to a reduced need for non-photochemical quenching near the top of the leaf, while providing more excitation energy to cells near the bottom of the leaf, and also to the interactive effect of the light spectrum and PPFD on photosynthesis [30].

## 4. Impact of Light Intensity on Photosynthesis

Light is one of the prominent factors necessary for plants to synthesize food and regulate various developmental processes [31]. Still, exposure to high or low levels of light is a major stress factor with a massive effect on the development of plants. High light acts as a stress factor due to an excess absorption of light during photosynthesis compared to the optimal requirement for the plant [32]. Similarly, exposure to low light brings differences in the pigment composition and reduces the stomatal conductance of the plant, which finally results in a reduction in photosynthetic performance [33].

### 4.1. High Light

Absorption of excess light results in the generation of harmful oxygen radicals, and this results in a process called photo-inhibition, which directly leads to a reduction in the primary productivity of plants. In plants subjected to high-intensity light, the photosynthesis rate is reduced due to processes such as photo-inhibition, photo-oxidation, photo-inactivation, photo-lability, solarization, and photodynamic reactions [34]. Photo-inhibition can be defined as the inhibition of the photosynthetic capacity of plants due to excessive light [32]. Photo-inhibition effects are often perceived when light energy surpasses the photosynthetic capacity. During high light stress, decreases in the quantum efficiency and photosynthetic rate have been observed, which are followed by damage to the photosynthetic apparatus resulting in the functional failure of PSII reaction centres and an increased dissipation of heat [35,36,37]. It has been proven that, under high light intensity, a very high oxidizing potential exists inside PSII reaction centres, which damages the key D1 core proteins. D1 protein turnover is an important part of photosynthesis recovery from stress damage, but the rate of D1 protein degradation is higher than that of its synthesis as stress intensifies; hence, PSII reaction centres will become photo-inactivated [38]. According to Faseela and Puthur, high light intensity causes a reduction in photochemical efficiency, followed by a high emission of dissipated energy in the form of fluorescence or heat, leading to damage of PSI and PSII. In the study, three varieties of *Oryza sativa*, Harsha Kanchana, and Mattatriveni were exposed to 2000 μmolm^−2^ s^−1^ light for 2, 4, 6 and 8h with the help of 1000W PAR64 metal halide lamps (Philips, Netherlands).

Various other important parameters, such as the maximum quantum efficiency of photosystem II, photochemical efficiency, electron transport flux, and chlorophyll/carotenoid ratio, are also negatively affected by high light stress [5]. Similarly, high light stress combined with extreme temperature leads to the photo-oxidation of chlorophyll [39]. Different environmental factors not only affect photosynthetic efficiency but also impart a negative effect on the rate of transpiration and stomatal conductance [40]. Photo-inhibition directly inhibits the quantum yield of photosynthesis and PSII photochemistry, directly affecting the fluorescence ratio F_V_/F_M_; to screen for this effect of repetitive light pulses on the photochemical reactions, wheat plants were exposed to 15,000 µmol photons m^−2^ s^−1^ [41]. The effect of high-intensity light causes a decline in the photosynthetic capacity of plants, and it can be directly analysed by the changes in PSII via measurements of the Chl *a* fluorescence parameter [42,43]. According to Faseela and Puthur, significant decreases in PI_ABS_, F_M_, SFI_ABS_, F_V_/F_O_, area above the fluorescence curve, Φ(Eo), and Ψo were observed under 0–8 h of high light treatment in different varieties of rice. Finally, it was concluded that short-term high light exposure was adequate for studying the effect of photo-inhibition in rice varieties and to characterize them as tolerant and sensitive varieties [44]. The higher decrease in the area above the fluorescence curve suggests that high light stress highly inhibits the electron transfer rates at the donor side of PSII, and it could induce structural damage to the reaction centres, leading to decreased excitation energy transfer from the antenna to the reaction centres, which ultimately leads to a drastic reduction in photochemistry in rice seedlings [43]. Under high light treatment, the electron transport quantum yield Φ(Eo) and the yield of electron transport per trapped exciton (Ψo) are greatly decreased, proving that high light exposure negatively influences the electron transport at the acceptor side of PSII. Similarly, high light stress causes significant alterations in F_O_, F_M_, F_V_, F_V_/F_M_, tF_M_, and the photochemical efficiency of PSII in various plant species [45]. Likewise, high light treatment decreases the rate of F_M_ in rice leaves; the maximum rate of reduction was recorded upon imparting 8 h of high light stress, and it indicated that the donor side of PSII, especially the oxygen-evolving complex, was damaged, which resulted in a reduction in the electron transport [5]. The decrease in electron transport activity can also be correlated with an increase in the lipid peroxidation of thylakoids under high light. Thus, it seems likely that the integrity of thylakoid membranes imparted by the intactness of the lipids is important for efficient photosynthetic electron transport activity, and under high-light-induced photo-inhibition, a loss in activity is registered as the thylakoid lipids are degraded [46].

Plant vitality as assessed by PI_ABS_ is the product of three independent characteristics: the concentration of the reaction centre and parameters related to primary photochemistry and electron transport [47]. According to Faseela and Puthur, treatment with high light impaired the PSII structure and electron transport system of sensitive rice varieties more severely than that of tolerant varieties, as inferred from the reduction in the values of the parameter SFI_ABS_. This reduction further results in a reduced rate of light trapping and electron transport in sensitive rice seedlings [5]. Similarly, the photosynthetic efficiency of two barley cultivars, Arabi Aswad and Arabi Abiad, was negatively influenced after high light exposure as concluded by various Chl *a* fluorescence parameters, and it was found that the former was more tolerant while the latter was more sensitive to high light stress [48]. For conducting of this experiment, barley seedlings were exposed to 1800 (µmol photon m^−2^ s^−1^) using a Philips high-pressure sodium lamp (600 W/230 V, 90.000 Lm). Further, the authors concluded that PI_ABS_ is a sensitive parameter to explore the effect of high light exposure on PSII activity after imparting stress.

An alteration of PSII energy fluxes per CS_O_ in response to high light stress in rice was visualized by phenomenological leaf models of the photosynthetic apparatus, and it was found that a sensitive rice variety (Swarnaprabha) had lower levels of calculated ETo/CS_O_ and RC/CS_O_ and higher values of DIo/CS_O_ and ABS/CS_O_ as compared to a tolerant variety (Aathira) after exposure to various extents of high light irradiation. There was no significant change observed in trapping flux (TRo/CS_O_) during high light treatment in either rice variety. Similar findings were also noticed in *Alhagisparsifolia* [49] and in *Camellia* leaves [50], where ETo/CS_O_ and RC/CS_O_ was reduced while DIo/CS_O_ and ABS/CS_O_ was enhanced upon exposure to high light stress. The high susceptibility of Swarnaprabha seedlings to high light exposure negatively influenced the PSII energy fluxes per reaction centre (RC) as revealed in the specific membrane model of PSII. The values of ABS/RC can be taken as a calculated average amount of chlorophyll that channels excitation energy into the reaction centres [47]. Therefore, the reduction in ABS/RC can be taken as the decrease in the average antenna size of the rice plants after exposure to high light irradiations. TRo/RC represents the maximal rate by which an exciton is trapped by the RC, resulting in a reduction in Q_A_, and a decrease in this TRo/RC indicates that all the Q_A_^−^ has been reduced but is not able to oxidize back due to further hindrance in the electron transport. As energy is not utilized for electron transport, it is lost through dissipation without any effective utilization.

According to Faseela and Puthur, energy dissipation, as indicated by DIo/CS_O_ and DIo/RC, increased in high-light-treated rice varieties, and dissipation occurs as fluorescence and energy transfer to other systems [44]. As the inactive reaction centres increased, the DIo/CS_O_ and DIo/RC also increased because the inactive centres were unable to trap the photons [51]. Indeed, high light treatment studied in different plants led to the conclusion that PI_ABS_, Φ(Eo), ETo/CS_O_, DIo/CS_O_, ETo/RC, and DIo/RC were found to be more reliable for exploring the effect of changes in PSII activity and are important for assessing high light tolerance potential. The enhancement in the rate of inhibition in photosynthesis under high light exposure could be due to the reduced carbon assimilation and inhibition of PSII photochemistry (both donor and acceptor sides of PSII) as evidenced by the F_V_/F_O_ values and the area over the fluorescence curve, respectively [47]. The high-light-induced changes in different Chl fluorescence parameters are compiled in Table 1.

### 4.2. Low Light

Another important variable impacting plant growth is low light. Low light interferes with the normal photosynthesis of plants by influencing photosynthetic pigments and their synthesis [57]. Under low light conditions, plants frequently acquire features, such as increased leaf area and chlorophyll content, that enable them to capture more optical energy [58]. Research on nine-bark also reveals the same result. The chlorophyll levels in the leaves of two species of nine-bark (*P. amurensis* and *P. opulifolius*) increased in response to low light, but the ability for photochemical activity of PSII and carbon assimilation significantly decreased [58].

Chl *a* fluorescence and photosynthesis are significantly affected by low light during leaf development. To examine this, two genotypes of sweet pepper, ShY and 20078, were treated with a low light intensity (75–100 μmol m^−2^ s^−1^) [59]. The net photosynthetic rate (Pn), chlorophyll content, carboxylation efficiency (CE), and photosynthetic apparent quantum efficiency (Φ_i_) in the sweet pepper leaves gradually increased in the early stages of vegetative growth and later dropped when subjected to low light levels. However, the time to reach peak values for all of the above characteristics was delayed in low-light-grown leaves. Under low light conditions, the quantum yield (Φ) of PSII was reduced in the sweet pepper, accompanied by an increase in NPQ in young and old leaves compared to mature leaves. The significance of the correlation between Φ_i_ and F_V_/F_M_, as well as Φ_i_ and PS II, was very low. However, the significance of the correlation between Φi and CE was much higher in the sweet pepper. Thus, rather than light reaction efficiency, the low-light-induced drop in photosynthetic efficiency throughout the latter stages of leaf growth was discovered to be regulated by Calvin-cycle carboxylation efficiency. The study found that plants could not maintain a greater P_N_ and lower Q_A_ and Φ_PSII_ for prolonged periods of time when exposed to low light, making them more sensitive to low light stress [59].

The effect of sub-optimal illumination was demonstrated by a study on three plant species: lettuce (*Lactuca sativa*), green amaranth (*Amaranthus hybridus*), and tree stonecrop (*Sedum dendroideum*). A comparison of the C3, C4, and CAM systems functioning in these three plant species under low illumination was also done in this study [33]. Low light treatment increased the pigment content, as it has in previous investigations. Similarly, at low light conditions, the maximum possible photosynthetic efficiency was around 0.8 units for all species and treatments, although the quantum yields of photophysical disintegration remained around 0.2 for all species. However, the fluorescence ratios remained constant after being corrected by light reabsorption processes, implying that photosystem stoichiometry was preserved. *L. sativa*, a C3 plant, demonstrated a decrease in photosynthetic efficiency as well as a significant increase in LHC size as a result of low light acclimatization. Low light treatment had no effect on *A. hybridus*, a C4 plant, while it lowered the antenna and improved the quantum yield of primary photochemistry in *S. dendroideum*, a CAM plant [33].

**Figure 4:**
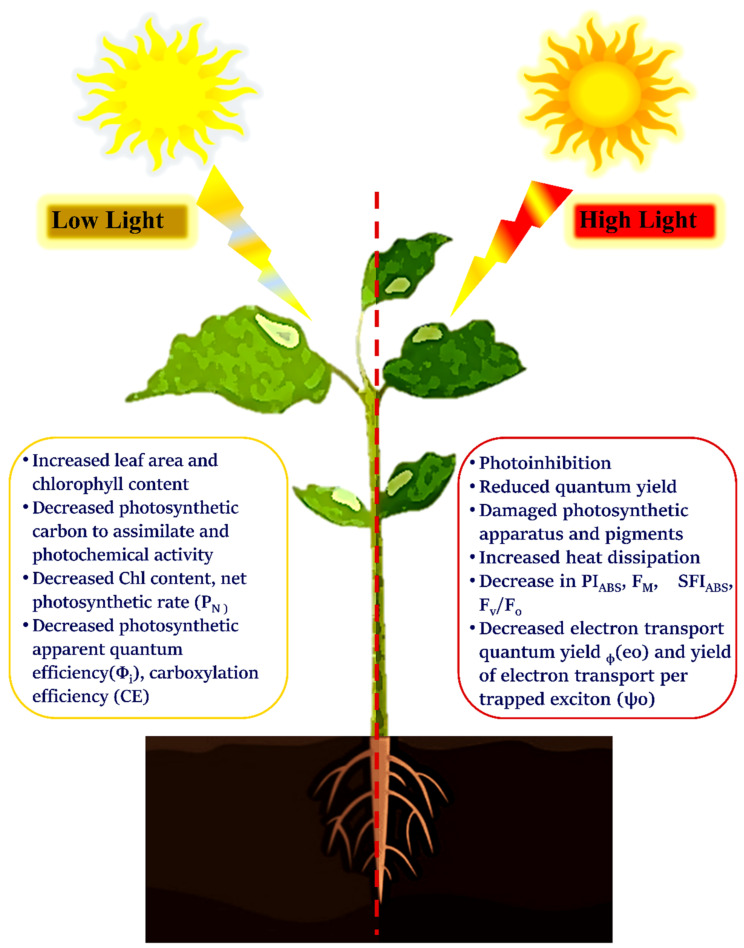
Effect of low and high light in the photosynthetic performance of plants.

Low light, on the other hand, has a positive effect on the photosynthesis and chlorophyll fluorescence in marine algae. The algae’s photosynthetic apparatus degrades in the presence of high light and other stresses, such as salinity, resulting in a decrease in photosynthetic efficiency. Low-light-illuminated algae, on the other hand, increased their photosynthetic efficiency from 0.2 to 0.4 [60]. A study on *Nannochloropsis oculate* yielded similar results. *N. oculates* was grown under two light regimes, high light (300 mol photon m^−2^ s^−1^) and low light (20,300 mol photon m^−2^ s^−1^), with a 12 h light–12 h dark cycle. In terms of chlorophyll fluorescence, the functional absorption cross-section of photosystem II (which is used to measure system-specific chlorophyll fluorescence absorption), NPQ, and absolute electron transfer rates ETR were all wavelength-dependent in both high light and low light cells [61]. Various changes in chlorophyll *a* fluorescence induced by high light and low light are represented in Figure 4.

## 5. Quality of Light

**Figure 5:**
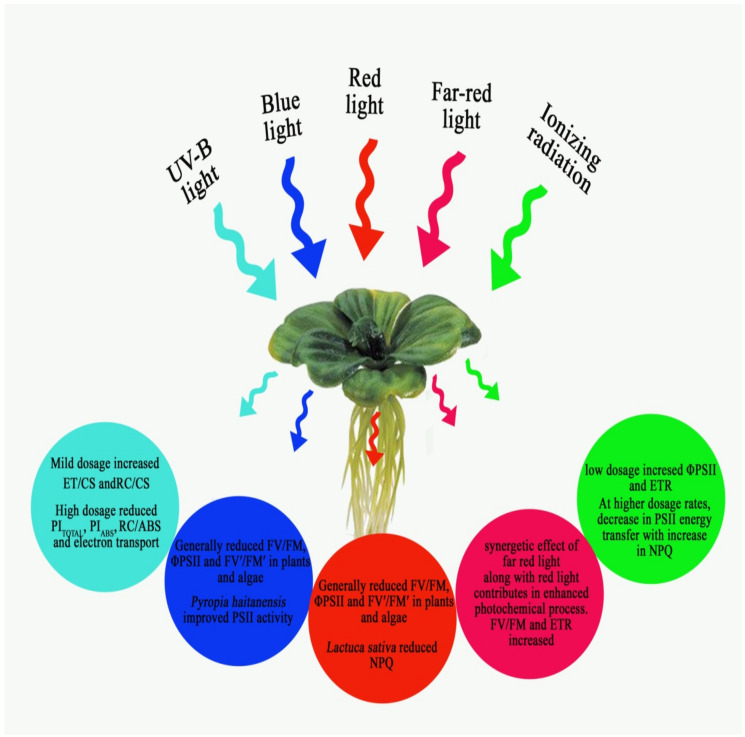
Impact of the spectral characteristics of light in the photosynthetic performance of plants.

Comparable with the intensity of light, the quality of light is also significant for normal photosynthetic processes in photoautotrophic organisms. The majority of plants show photosynthetic activity in photosynthetic active radiation (PAR) ranging from 400–700 nm [62]. In autotrophic organisms such as plants and algae, on exposure to a specific wavelength of PAR, the chlorophyll fluorescence parameters change according to the modification of the photosynthetic performance of these organisms. The interpretation of the chlorophyll *a* fluorescence curve and the associated JIP parameters are considered an efficient and non-invasive tool to analyse the impact of exposure to a specific spectrum/monochromatic light. Simultaneously, the combinations of different qualities of light should influence the chlorophyll *a* fluorescence response in plants. *Cunninghamia lanceolata* exposed to different ratios of red, blue, green, and purple induced significant modifications in the electron transport efficiency by modifying the F_V_/F_O_, F_V_/F_M_, coefficient of photochemical (qP) and NPQ quenching, and relative rate of electron transport [7]. Various changes in the chlorophyll a fluorescence parameter induced by spectral modifications of incident light are represented in Figure 5.

### 5.1. UV Radiation

UV light is typically classified into three wavelength ranges: UV-A (315–400 nm), UV-B (280–315 nm), and UV-C (200–280 nm). In that, UV-B has a significant impact on almost all organisms, although it is a very small part of the light spectrum. UV radiation below ∼295 nm will be absorbed by the stratospheric ozone layer, and the rest that have comparatively high energy will cause damage to DNA and other cellular processes [63]. Moreover, a low dosage of UV-B with peaks at ∼295 to 300 nm will induce specific photomorphogenic responses, such as flavonoid biosynthesis, hypocotyl growth suppression, increased photosynthetic efficiency, and enhanced production of photosynthetic pigments [63,64,65].

When seeds of kidney bean varieties, beets, and cabbage were subjected to a low dosage of UV-B (760 mW/cm^2^ for 90 min), enhancements in the plastid pigments, anthocyanins, and carotenoid contents were observed [66,67]. The microalgae *Chlorella* was subjected to two different short-term UV treatments: (I) 8.54 wm^−2^ UV-A and (II) 8.54 wm^−2^ UV-A + 1.17 wm^−2^ UV-B, both for 5 h. No significant change in chlorophyll *a* or carotenoid content occurred, and it may be because of the short term of the treatment. Moreover, the strain that was subjected with both UV-A and UV-B had a significant reduction in F_V_/F_M_ and rETR_m_ (maximum relative electron transport rate) compared to UV-A alone. The F_V_/F_M_ reduction indicates a decrease in the photosynthetic competency of PSII. A decrease in the electron transport rate and photosynthetic apparatus degradation were indicated by the reduced rETR_m_ values. This reduction in the electron transport rate can be due to the disfunction of the oxygen-evolving complex or cytochrome *bf* [68]. Low UV radiation generally does not cause any lethal effects to plants. When low-dosage UV-B (7.1 kJ m^−2^ d^−1^) was imparted on *Glycine max* seedlings, no significant change has observed in the chlorophyll content, carotenoid, F_V_/F_M_, or F_V_/F_O_ [69].

In an experiment conducted on rice by Thomas et al., UV-B at 4–6 kJ m^−2^ d^−1^ increased both the total chlorophyll and carotenoid contents. Another interesting finding from this study was that rice plants treated with low UV-B and subjected to various stressors, such as NaCl and high UV irradiation, exhibited enhanced total chlorophyll and carotenoid contents compared to untreated plants. From this, it was evident that low UV-B can enhance stress tolerance. In addition, it was clear that UV-B can also influence photosynthetic efficiency according to its dosage. The dynamic energy pipeline leaf model expressing the energy flux parameters for electron transport per unit of sample area, trapping of excitation energy, and light absorption from Chl *a* fluorescence was derived in the case of rice imparted with 4–6 kJ m^−2^ d^−1^ UV-B; it was seen that parameters such as density of active reaction centres (RC/CS) were enhanced, which in turn increased the activity of the reaction centres. UV-treated plants also exhibited a reduced dissipation of energy, which led to an increase in photon absorption, electron transport, trapping of photons, and electron transport flux per reaction centre (ET_O_/CS_M_) [70]. However, like other stresses, high-dosage UV-B (28 kJ m^−2^ d^−1^) can cause a reduction in and degradation of chlorophyll and the associated accessory pigments.

Furthermore, a significant decrease was observed in the flux of absorption (ABS/RC) and electron transport flux (ET_O_/RC), and at the same time, an increase in dissipated energy (DI_O_/RC) was recorded when rice seedlings were subjected to high-dosage UV-B (28 kJ m^−2^ d^−1^) [5,71]. Another interesting fact about UV radiation is its ability to alter the activity of both PSI and PSII. As mentioned earlier, a low intensity (4–6 kJ m^−2^ d^−1^) of UV imparted to rice enhances the activity of PSI and PSII. Moreover, a significant increase in PSI efficiency was noted in this experiment. However, when the intensity of UV-B increased to 21–28 kJ m^−2^ d^−1^, a greater reduction was observed in the case of PSII activity. This reduction was mainly due to the degradation of PSII under UV stress conditions. UV radiation primarily damaged the D1/D2 reaction centre proteins, the oxygen-evolving complex, and other components on both the acceptor and donor sides of PSII. In contrast to PSII, PSI activity in the rice seedlings was greatly enhanced by UV-B exposure (5, 65).

UV-B’s negative influence has been demonstrated; when three-year-old Scots pine seedlings were treated with 2.88 kJ m^−2^ d^−1^ for 8 h per day for two days, the chlorophyll fluorescence transient (OJIP) curve showed a significant change. Compared to control, UV-B-treated seedlings displayed considerably delayed fluorescence, and the I–P amplitude was much lower. Photosynthetic performance indexes (PI_TOTAL_ and PI_ABS_), Ψ0/(1 − Ψ0), ϕP0/(1 − ϕP0), δR0/(1 − δR0), RC/ABS, and electron transport from Q_A_^−^ to end electron acceptors at the PSI acceptor side (RE_o_/RC) significantly decreased due to the UV-B treatment. On the other hand, the antenna size of an active reaction centre, the flux of electrons transferred from QA to PQ per active reaction centre, and the flux of energy dissipation in processes other than trapping per reaction centre all significantly increased [72]. The deleterious effect of UV-B was again observed when one-month old seedlings of *Scutellaria baicalensis* were subjected to UV-B at 10.30 kJ m^−2^ d^−1^ for 15 days; P_MAX_ and ETR_MAX_ were considerably reduced, but no significant change was observed in F_V_/F_M_ [73]. Modulations in the photosynthetic responses of basil (*Ocimum basilicum*) were prominent under UV-B exposure, and the responses were different in green and purple basil. The fluorescence parameters F_V_/F_M_ and PI_ABS_ were significantly reduced in green basil but unaltered in purple basil, and similar responses were observed in TR/CS and ET_O_/CS. However, the dissipation was higher in purple basil, indicating the efficiency of this plant to tolerate the higher UV-B exposure by dissipating the excess energy as heat [74]. Three-year-old Scots pine seedlings illuminated with UV-B radiation at 309 nm had prominent enhancements in the antenna size of an active reaction centre (ABS/RC) and the dissipation energy. Moreover, UV-B radiation reduced the efficiency of the oxygen-evolving complex (OEC), which is related to the OJIP parameter V_K_/V_J_, and this resulted in the inefficiency of electron transport for photochemical reactions [72].

In a recent study, *phy A* and *phy B* mutants of *A. thaliana* were imparted with 311 nm UV-B for 1 h, and this caused a reduction in photosynthesis, F_V_/F_M_, respiration rates, and PI_ABS_ in all plants, but the *phy B* mutants exhibited a decrease in photosynthetic activity. Moreover, this was due to the mutant’s lower content of carotenoids and UV-absorbing pigments [75]. From all these studies, it is evident that UV radiation can cause specific photomorphogenic responses and certain deleterious effects, especially in reducing the photosynthetic pigments and photosynthetic efficiency in plants. It all depends on the intensity of UV radiation and the plants receiving the irradiation.

UV-A is well-known for its high penetration power, but UV-A, along with red light, induces certain positive changes in photosynthetic efficiency. It was demonstrated that, when 14-day-old seedlings of *Lathyrus sativus* L. were subjected to UV-A (315–400 nm): red light (ratio 10:90, 110 μmol m^−2^ s^−1^), no significant change was found in Chl *a*, Chl *b*, total chlorophyll, or carotenoid contents. Even though the stomatal conductance and transpiration rate remained unchanged, the photosynthesis rate significantly increased [76]. In chlorophyll fluorescence studies, it was revealed that the F_V_/F_O_ (donor side of PSII- OEC) value remains unchanged, and at the same time, significant increases in φ_Eo_, ψ_Eo_, and the electron transport flux were observed. On the other hand, a significant enhancement in S_M_ (total electron carriers per RC) and a decrease in DI_O_ were found. According to the observed data, the proper maintenance of effective OEC and enhanced total electron carriers resulted in efficient linear electron transport, which contributes to an increase in CO_2_ carboxylation efficiency [77,78,79,80].

From all the above-mentioned studies, it is evident that chlorophyll fluorescence is an efficient tool to assess the impact of UV radiation in plants. Moreover, UV radiation can induce both positive and negative changes in the photosynthetic efficiency of plants. In addition, these changes mainly depend upon the intensity of incident UV radiation and the UV tolerance potential of the plant. UV-radiation-induced changes in different chlorophyll *a* fluorescence parameters are compiled in Table 2.

### 5.2. Ionising Radiations

Given that the environment is inextricably subjected to natural and manmade radiations, it is necessary to examine the radiation-induced response of plants. Ionising radiations (IR) have been shown to have both good and detrimental effects on a variety of plants [82]. Numerous new studies on the effect of IR on photosynthesis and related topics have been published.

Photosynthetic pigments in plants are significant IR targets. For clear evidence, *Pinus strobus* and *P. sylvestris* were maintained in a radioactive waste disposal area (Welcome Residue Site, Port Hope, Ontario) and exposed to a gamma dose rate of 10.15 mR/hr. This IR radiation altered the pigment concentration and ratio in a dose-dependent manner. As with any other stress, modest IR doses induce the chlorophyll content to remain constant or increase. The chlorophyll and carotenoids content will decrease as the IR dose increases, which also increases the Chl *a/b* ratio [83]. The synthesis pathway for chlorophyll *b* is more IR-resistant, and Chl *b* predominates in PSII’s external antenna and light-recovery complex. In this case, IR mostly influences chlorophyll content by reducing the quantity and size of chloroplasts [84,85]. In addition, NPQ fluctuates in response to different IR dosages. The dynamics of unregulated NPQ rise at high IR dosages and diminish at low IR doses [85,86]. The rate of electron transport and the quantum yield of PSII are crucial measures of the efficiency of primary photosynthetic processes. Acute irradiation at high doses induces a quick drop in PSII-Y (PSII) and ETR quantum efficiency, but low doses have a stimulatory impact [86,87]. F_V_/F_M_, which indicates photosynthetic performance, is usually IR-resistant; however, it varies depending on the electron transport rate. F_V_/F_M_ can drop or slightly rise during irradiation, depending on the dose and kind of IR [84].

The capacity of photosystem II was not affected by low-dose gamma (γ) treatment in Arabidopsis thaliana. Plants balance photosynthesis in low light conditions by boosting PSII efficiency and maximal electron transport rate (ETR_MAX_) while decreasing nonphotochemical quenching [82]. Short-term gamma radiation reduces the photosynthetic capacity and creates reactive oxygen species in the algae *Chlamydomonas reinhardtii*, according to a study. Gamma radiation has an effect on PSII photochemistry in algal photosynthesis at higher dose rates, with light energy dissipating predominantly through non-photochemical processes, presumably as a defensive mechanism against ROS generation and oxidative damage. At higher dosage rates, a decrease in PSII energy transfer was found, most likely due to ROS generation caused by gamma radiation, which clearly indicates the dose-dependent inhibition of photosynthesis [85]. Another study on *Capsicum* also proved that high dosages of ionising radiations can act as stress, limiting photosynthetic parameters, whereas at a low dosage, it can act as a stimulant [88].

### 5.3. White Light Exposure

**Figure 6:**
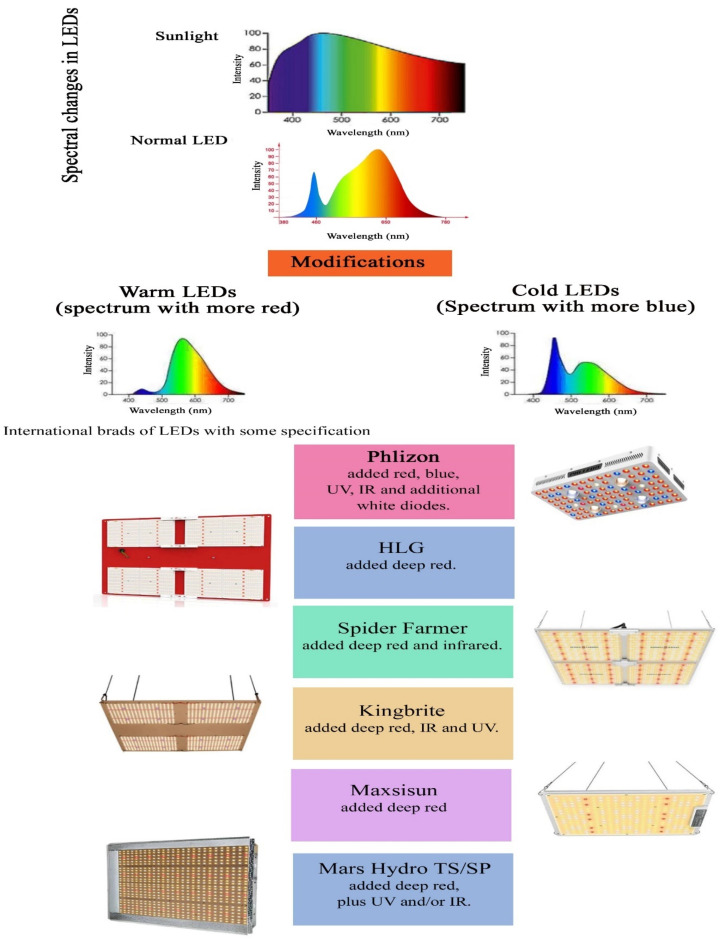
Spectral variations in LEDs from popular brands with major specifications.

Generally, the energy source of photosynthetic processes is sunlight, which is white with all the different wavelengths of light. Instead of sunlight, the utilization of artificial light sources, such as light-emitting diode (LED), aid the optimization of photosynthetic efficiency by regulating the photo flux [89]. These LED lamps have a high PAR efficiency with a minimal increase in the temperature compared to high-intensity discharge (HID) lamps [90]. White light is made up of different colours, and exposure to white light favours photosynthesis under controlled conditions. LEDs have the potential to emit white light with different spectral characteristics, and different industries have focused on developing LEDs with different ratios of blue and red emission. Different LEDs with varying light-emitting features are represented in Figure 6.

An analysis of the polyphasic Chl *a* fluorescence (OJIP) transients of *Rosa hybrida* cv. ‘Avalanche’ indicated the efficiency of white light over monochromatic illuminations [91]. In addition, plants exposed to white light showed a higher *F*_0_ and *F*_J_ compared to the plants exposed to red light. ABS/RC and TRo/RC were higher in white-exposed plants compared to specific-spectrum exposure (red and blue light), but the energy move to the electron transport chain (ETo/RC) was minimal under the exposure to white light. As the energy utilization is very low under white light, the absorbed portion of energy will dissipate, which is represented by the increased DIo/RC related to red and blue light [91]. It was found that the efficiency of white light increased when combined with red or UV light, which enhanced the photosynthetic efficiency of a plant by altering the leaf pigment composition and leaf area [92]. In a study conducted on purple cabbage, the Pn value was higher in plants exposed to white light compared to those exposed to green, yellow, red, and blue light. Moreover, the PI_ABS_ was insignificantly modified on monochromatic light exposure [93]. The white light exposure maintains the photosynthetic efficiency and development of plants better than any other monochromatic light exposure, and chlorophyll fluorescence responses also support these findings [93].

### 5.4. Red and Far-red Light Exposure

Among the photosynthetic active radiation, red light composed of a spectral wavelength ranging from 600–680 nm and far-red light with a wavelength above 700 nm serve as vital cues for plant development. Various studies revealed that irradiation with a monochromatic beam of red and far-red light significantly altered the photosynthetic performance of plants. Changes in the photochemical activity were also reflected in the Chl*a* fluorescence parameters. Studies on several plant species have reported the physio-chemical modulations induced under red and far-red light, which are well-documented in several plant species, such as cucumber, lettuce, tobacco, and *Aradidopsis* [57,94,95]. Even though red light can result in the highest quantum yield of CO_2_ fixation, supplementing red light alone inhibits plant growth [96,97].

When *Cucumis sativus* plants were grown under lights of different quality (purple, blue, green, yellow, red, and white light), all with the same photosynthetic photon flux density (PPFD) of 350 μmol m^−2^ s^−1^ for 5 days, the maximum plant growth occurred in the case of plants irradiated with white light. In this study, it was observed that upon irradiation, F_V_/F_M_, ΦPSII, photochemical quenching coefficient qP, and the efficiency of excitation captured by open PSII centre (F_V_′/F_M_′) were more reduced in plants exposed to red light than in plants exposed to white light, and an increase in NPQ corresponding to the dissipation of energy and a reduced photosynthetic efficiency were recorded under red light [94]. *Calendula officinalis* grown in red light showed a reduction in F_V_/F_M_ and performance index, and at the same time, there was an increase in quantum yield of energy dissipation; likewise, the fluorescence intensities in the OJIP phases also increased [98]. Evidence of photosystem damage that occurred during exposure to pure red light in *C*. *sativus* was reported by Trouwborst et al. in 2016 [95]. In the experiment, plants were exposed to red light with a PPFD of 100 ± 5 μmol m^−2^ s^−1^ for 16 h, and there was a reduction in F_V_/F_M_ and photosynthetic capacity (A_max_). They used an LED of 100% red light (Li-190, LiCor inc., Lincoln, NE, USA). In another study, when *Lactuca sativa* plants were irradiated with 200 μmol m^−2^ s^−1^ light for 6 h with six different combinations of red/blue (R/B) ratios, the results showed that the red and blue light combinations were more efficient than the monochromatic light alone. Photosynthetic rate increased with the decreasing ratio of R/B, and monochromatic exposure to red light had the lowest Pn and A_max_ reported from all these treatments, because irradiance with red light alone inhibits electron transport from PSII to PSI and thereby reduces the efficiency of plants grown under red light only. It was also noted that R/B and monochromatic red treatments have maintained the highest quantum efficiency of PSII [95]. According to Kim et al., the combined treatment of red and blue lights in chrysanthemum plants enhanced the chlorophyll content of the plants [90].

Studies have shown that the synergetic outcome of far-red light along with red light contributes to an enhanced photochemical process and overcomes the adverse effect caused by monochromatic red light, called red light syndrome [4]. The exposure of *Lactuca sativa* to red LED (54 W; Popular Grow, Shenzhen Houyi Lighting, Shenzhen, China) and far-red LED (Epistar, Hsinchu, Taiwan) showed a 6.5% enhancement in the quantum yield of PSII and a reduction in NPQ; thereby, an overall increase in net photosynthesis was noted [99]. In *Arabidopsis thaliana*, 10–15 min of exposure to fluctuating light (intermittent exposure to high light/low light at 800/30 μmol m^−2^ s^−1^) with far-red light increased the photosynthesis [57]. In another study, exposure of the leaves of lettuce plants to different R/FR LEDs altered the photochemistry of the plants. As compared to the control, which had an F_V_/F_M_ value of 0.81, there occurred a reduction in the value of F_V_/F_M_, which was less than 0.8 after 11 days of treatments; the results indicate that in lettuce, R/FR exerts a stress on the plants, and this may be due to the absence of blue light [100]. The quantum yield of photosynthesis, ETR, and photochemical quenching were increased when seedlings of tomato were irradiated with a low R:FR value compared to the seedlings irradiated with a high R:FR value [3].

### 5.5. Blue and Green Light

For plant growth, blue light is a crucial factor as it is involved in photomorphogenesis and photoperiodism. Moreover, blue light has a significant role in the determination of the photosynthetic efficiency of plants [30,101]. The chlorophyll *a* fluorescence curves of *Ilex acuifolium*, *Piceaabies*, *Phaseolus vulgaris*, *Zea mays*, *Fagus sylvatica*, and *Nicotiana tabacum* were transformed on exposure to blue light. Irradiation with blue light induced two shoulder peaks in the fluorescence spectra of all six plants at 690 and 730 nm [101]. *Lactuca sativa* exposed to blue light at 450 nm with an intensity of 400 μmolmol^−1^ for 24 h showed a significant reduction in photosynthetic efficiency as compared to the plants exposed to white radiation [102]. In this study, the authors focused on the stomatal conductance, leaf structure, and carbon fixation efficiency. In addition, they interpreted PSII efficiency using chlorophyll *a* fluorescence by analysing F_V_′/F_M_′ and Φ_PSII_. F′ is different from the value of F, where F′ is obtained in experiments using the exposure of multiple saturation pulses. This parameter aids in differentiating photochemical and non-photochemical quenching. In other words, after strong actinic light exposure, the actions of photochemical and non-photochemical events result in the quenching of energy; the quenching or deexcitation of PSII molecules takes almost 20 min after the first pulse and then reaches a steady-state level of fluorescence. F′ indicates this steady state of fluorescence in the illumination [29]. The parameter F_V_′/F_M_′ indicates the efficiency of PSII photochemistry on exposure to light when all the reaction centres are in an oxidized state [29]. On blue light exposure, this parameter showed a significant reduction in *Lactuca sativa.* The effective quantum yield of PSII, Φ_PSII_, was also decreased on exposure to blue light, and the response of F_V_′/F_M_′ and Φ_PSII_ strongly indicate that continuous blue light radiation reduces the efficiency of PSII, resulting in a reduction in the photosynthetic rate.

In contrast, a comparison of the photosynthetic efficiency in *Betula pendula* irradiated with blue and red light showed that blue light significantly increased PAR and chlorophyll biosynthesis [103]. When different proportions of blue light (0, 7, 15, 22, 30, 50, and 100%) radiation were provided to *Cucumis sativus*, the impact of blue light on photosynthesis was clearly visualized [103].

*Cucumis sativus* irradiated with 100 ± 5 μmol m^−2^ s^−1^ blue light at 450 nm for 16 h showed a gradual increase in PSII efficiency on exposure to 0–50% proportions of blue light. F_V_/F_M_ and Φ_PSII_ were analysed to estimate the efficiency of PSII photochemistry, and both showed an increase [104]. However, on 100% blue light exposure, the photosynthetic efficiency of *Cucumis sativus* was decreased. In the same study, the fluorescence images of leaves were made with the help of a PSI Fluorcam 700MF Chl fluorescence imaging system. This image was used to record the distribution of F_V_/F_M_ on the leaves under blue light exposure, where 30% blue light showed uniform F_V_/F_M_ distribution, whereas the 0% blue light showed a heterogeneous F_V_/F_M_ distribution in the veins and lamina. Besides the spectrum, the source of irradiance also affects the photochemical reactions of plants, which was analysed in *Rosa* × *hybrida* [105]. When this plant was irradiated with different blue (B) light combinations generated by light-emitting diodes (LED, high B 20%) and high-pressure sodium (HPS, low B 5%) lamps, it showed differential photosynthetic responses towards the irradiations.

Plants exposed to LED with a high blue irradiance ratio increased their chlorophyll biosynthesis, stomata conductance, and photosynthetic performance. F_V_/F_M_ and Φ_PSII_ showed an increase on exposure to LED light, indicating an improved efficiency of PSII activity. From this study, it was clear that if plants were exposed to monochromatic blue light, it results in the reduction in photosynthesis, but by increasing the blue light proportion in the PAR, the photosynthetic efficiency could be improved. In *Cucumis* *lanceolata* and *C. sativus*, the reduction in Φ_PSII_ was correlated to the reduction in photochemical quenching, indicating an inefficiency in the energy utilization due to monochromatic light exposure, which results in reduced photophosphorylation [7].

*Physcia aipolia* and *Xanthoria parietina* are phototrophic foliose lichens that show differential photosynthetic activities on blue light exposure. The thalli of *Physcia aipolia* and *Xanthoria parietina* showed significant reductions in F_V_/F_M_ and ΦPSII on blue light exposure of 650 μmol m^−2^ s^−1^ for 4 h [106]. Another alga, *Pyropia haitanensis* showed significant increases in PSII activity, electron transport, and photochemical quenching on exposure to blue light. Corresponding to the results, a drastic reduction in NPQ was observed, indicating the potential of these organisms in the efficient utilization of absorbed light energy [107].

Due to the inefficiency of their green light absorption, plants reduce the photosynthetic yield under green light exposure, but this green light can penetrate to the internal parts more efficiently [30]. *Physcia aipolia* and *Xanthoria parietina* showed differences in PSII activity on green light exposure [106]. On exposure to green light, *Pyropia haitanensis* maintained the photochemical quenching and quantum yields of PSII but failed to maintain the electron transport efficiency as compared to the control. Moreover, exposure to blue-green light increased the photosynthetic pigments of different unicellular marine algae, and the changes in the quality of light significantly altered the composition of pigments in this organism [108]. A study conducted on *Lactuca sativa* showed the importance of green light intensity in plant photosynthetic performance [109]. Different photosynthetic photon fluxes (PPFs) (100, 200, and 300 μmol m^−2^ s^−1^) of green light were used, and the plants irradiated with PPF 200 had an augmentation in their photosynthetic rate (Pn). The intensity of the photon flux of green light had a significant role in the photosynthetic performance; green light with a high PPF (1000 μmolm^−2^ s^−1^) increased the photosynthetic performance and quantum yield of CO_2_ assimilation in the leaves of *L. sativa* compared to the high-PPF blue light [30]. Therefore, it is evidenced that the spectral characters and photon flux have critical roles in the determination of photosynthetic performance in plants.

### 5.6. Combinations of Different Quality of Light and its Imprints on Photosynthetic Performance

The exposure of leaves to combinations of light with different spectral characters and varying intensity has the potential to modify the photochemical responses of a plant [104]. *Cunninghamia lanceolata* exposed to different light spectra in an in vitro culturing process rapidly changed the chlorophyll *a* fluorescence responses [7]. They used a light source with different ratios of red (R), blue (B), green (G), and purple (P). The different treatments were R/B; R/B/P; R/B/P/G; and W with proportions of 88.9% R+ 11.1% B; 80.0% R+ 10.0% B+ 10.0% P, 72.7% R+ 9.1% B+ 9.1% P+ 9.1% G, and white light (control), respectively. The parameters evaluated were F_V_/F_O_, F_V_/F_M_, NPQ, and relative rate of electron transport in the leaves of *C. lanceolata* plantlets. On exposure to R/B light, the photosynthetic efficiency of PSII, photolysis of water, and quenching activities were reduced, whereas the electron transport efficiency was maintained in the plant. On R/B/P exposure, the photochemical quenching coefficient showed a highly significant reduction, whereas the electron transport efficiency showed a drastic increase on exposure to R/B/P/G. This result indicates that the quality of irradiating light can cause significant modifications in the fluorescence parameters [7]. Another experiment conducted in *Cucumis sativus*, which was irradiated with different light sources with red, purple, blue, green, and yellow lights, showed a reduction in photosynthetic efficiency [110]. As compared to the plants grown in white light, all other plants had a reduced Φ_PSII_, i.e., the effective quantum yield specific to PSII [111]. Different combinations of red light (R) and blue light (B) were used in *Nannochloris oculata* to evaluate the growth responses, and the combinations were 0R7B, 1R6B, 2R5B, 3R4B, 4R3B, 5R2B, 6R1B, and 7R0B. Of these, 7R0B and white light induced the production of chlorophyll *a* and carotenoid pigments in this organism. The red light exposure created a limiting factor, as *N. oculate* is inefficient in utilizing it, and it demands excess biosynthesis of photosynthetic pigments, especially carotenoids [112]. Combinations of red and far-red light also induced alterations in the process of photosynthesis; *Mesembryanthemum crystallinum* was treated with two different red–far-red ratios, (R/FR = 1.2) and (R/FR = 0.8). In that study, both treatments reduced the net photosynthetic rate owing to the degeneration of photosynthetic pigments [110]. Red lettuce was exposed to a mixture of blue (B, 20%) and red (R, 80%) light with different intensities, and it was found that the increase in the intensity of light was directly related to the NPQ. However, the intensity of this combination insignificantly influenced the F_V_/F_M_ and the quantum yield of PSII electron transport [113]. It was very obvious that combinations of light are more effective for the betterment of photosynthesis than monochromatic light exposure [1].

The negative impacts of UV-B exposure on the photosynthetic reactions could be alleviated with exposure to blue light. This efficiency of blue light was explained in relation to the induction of phenolics biosynthesis, which inhibits photosynthetic pigment degradation [114].

## 6. Photosynthetic Responses Related to Duration of Light

The duration of the exposure determines the fluorescence response towards light by regulating the electron transport and CO_2_ fixation [30]. Visible light may cause photo-inhibition in plants. Long-term exposure to low PPF and short-term exposure to high PPF induce photo-oxidative damage in plants. Naturally occurring levels of visible light can cause photo-inhibition. *Lactuca sativa* were exposed to continuous visible light at 150 μmol m^−2^ s^−1^ and 50 μmol m^−2^ s^−1^ intensities for 7 d, intermittent light for 2 h at 150 μmol m^−2^ s^−1^ and 50 μmol m^−2^ s^−1^ intensities for 2 d, and 2 h dark for 5 days. The results showed that, in the plants that were continuously exposed to light for 7 d and in the plants kept under intermittent light, F_V_/F_M_ and performance index were reduced. This revealed that exposure to high light reduced F_V_/F_M_ and performance index irrespective of the duration of light [115]. The electron transport, thylakoid protein biosynthesis, and CO_2_ fixation were influenced by the duration of light in *Triticum aestivum*; intermittent light was provided for 1 day with high light (800 μmol m^−2^ s^−1^) followed by 3 days of low light (100 μmol m^−2^ s^−1^). *T. aestivum* exhibited a differential fluorescence response depending on the duration of the light exposure, and the photosynthetic responses of the plant were analysed for 15 min and 2 h durations. The quantum yield of PSII and NPQ were at a maximum in high-light-exposed (constantly) plants as compared to low-light- and intermittent-light-exposed plants. However, after 2 h of high light treatment, ΦPSII had a reduction, and NPQ was increased in all the treatments. Although the decrease in ΦPSII was prominent in low-light- and intermittent-light-exposed plants, the increase in NPQ was pronounced in plants exposed to high light [116]. Moreover, fluctuating light is able to reduce photo-inhibition reactions, which was proven in maize plants [117]. For this experiment, the plants were alternatively illuminated with high light (1600 μmol photons m^−2^ s^−1^) and low light (50 μmol photons m^−2^ s^−1^) at different frequencies. A rapid increase in the number of fluctuations of light resulted in a reduction in chlorophyll content and decreases in PSI and PSII activities, with a modification in the normal shape of the chlorophyll fluorescence transduction curve [117].

The duration or time of light exposure is a significant component in determining an area’s night-time habitat variety, and light pollution is defined as light exposure that has a negative impact on diversity. Artificial light pollution at night (ALAN) has the ability to disrupt the behaviour patterns of several animals, particularly coral reefs and their related algae. ALAN causes a reduction in electron transport, which is associated with the reduction in algal biomass, which has generated oxidative stress in corals [81].

## 7. Conclusions

Drastic variations in the quantity and quality of light are reflected in the reduced photosynthetic activity of photoautotrophs. Even small changes in light characteristics and duration of exposure affect photosynthesis in plants and algae. The versatility and quantity of chlorophyll *a* fluorescence data allow detailed interpretation for each stage of the light response. Of the various fluorescence parameters, FV/FM and performance index are the most common parameters explained exclusively by researchers to evaluate the effects of light stress, and performance index has been more efficient in detecting early reductions in photosynthesis. Plant production and algal biomass production depend on light characteristics; therefore, it is important to standardize the optimal light conditions. In this review, chlorophyll *a* fluorescence analysis is proposed as one of the best strategies to analyse the light conditions for plants to achieve maximum photosynthesis and growth.

## Figures and Tables

**Table 1:** Impact of high light exposure on the chlorophyll *a* fluorescence parameters of different plants.

Plant	Dose	Effect	Reference
*Oryza sativa* L.	2000 µmol m^−2^ s^−1^	Increased ABS and DI_o_/RC	[5]
*Chlamydomonas reinhardtti*	2000 µmol m^−2^ s^−1^	Decreased photosynthetic efficiency and increased photo-oxidative damage	[36]
*Dunellia salina*	1000 µmol m^−2^ s^−1^	Induced photo-damage to cells and increased free hydroxyl radicals	[40]
*Glycine max* (L.) Merr.	2000 µmol m^−2^ s^−1^	Decline in photo-chemical quenching coefficient and F_V_/F_M_	[52]
*Oryza sativa* L.	2000 µmol m^−2^ s^−1^	Reduction in photo-chemical efficiency accompanied by high emission of dissipated energy leading to PS I and PS II damage	[44]
*Lillium* sp.	2000 µmol m^−2^ s^−1^	Energy dissipation level increased DI and level of excess light absorbed by F_V_/F_M_	[53]
*Chlorella* sp.	2000 µmol m^−2^ s^−1^	Inhibited photosynthesis and ligh-dependent oxygen bleaching	[54]
Grapevine	2000 µmol m^−2^ s^−1^	Increased DI_o_/RC, reduced photosynthesis	[55]
*Arabidopsis* *thaliana* (L.) Heynh.	1000 µmol m^−2^ s^−1^	Degradation of chl b by an isozyme chl –b reductase	[56]
*Hordeum vulgare* L.	1800 µmol m^−2^ s^−1^	Severe damaging effect on F_V_/F_M_ and PI_abs_	[48]

**Table 2:** Impact of UV radiation on the chlorophyll *a* fluorescence parameters of different plants.

Plant	Intensity of Light	Response in Important JIP parameter	References
*Oryza sativa*	UV-B (4–6 kJ m^−2^ d^−1^)	RC:CS, ABS:CS, ET_o_:CSM, TR:CS, ET:CS increased	[70]
*O. sativa*	UV-B (28 kJ m^−2^ d^−1^)	ABS/RC and ET_o_/RC decreased; DI_o_/RC increased	[5]
*Pinus sylvestris* L.	UV-B (2.8 kJ m^−2^ d^−1^ for 2 days)	PI_Total_, PI_ABS_, RC/ABS, RE_o_/RC decreased; ABS/RC, ET_o_/RC, DI_o_/RC increased	[72]
*Scutellaria baicalensis*	UV-B (10.30 kJ m^−2^ d^−1^ for 15 days)	P_max_ and ETR_max_ decreased; F_V_/F_M_ no change	[73]
Microalgae *Chlorella* sp.	UV-A (8.54 wm^−2^) + UV-B (1.17 wm^−2^)	F_V_/F_M_ and rETR_m_ decreased	[68]
*Glycine max*	UV-A (7.1 kJ m^−2^ d^−1^)	F_V_/F_M_ and F_v_/F_o_ no change	[69]
*Wolffia arrhiza*	UV-B (4 wm^−2^)	φ_po_, φ_Eo_, φ_o_ decreased; RC/ABS and RC/CS increased	[77]
*Spinacia oleracea*	UV-B (50 µEm^−2^ s^−1^)	F_V_/F_M_ decreased	[78]
Cyanobacterium *Synechocystis* sp.	UV-B (2.4 wm^−2^)	F_max_ increased	[79]
*Spinacia oleracea*	UV-B (5 wm^−2^)	F_(t)_/F_o_ decreased	[81]

## Data Availability

All information is presented in this article.

## Abbreviations

**Phases in Induction Curve**	
O = F_O_	Minimal fluorescence/First step of chl *a* fluorescence transient
J = F_J_	Intermediate step in the chl *a* fluorescence transient at 2 ms
I = F_I_	Intermediate step in the chl *a* fluorescence transient at 30 ms
P = F_P_ = F_M_	Maximal fluorescence level/Final step of chl *a* fluorescence transient
K	Intermediate step in the chl *a* fluorescence transient at 0.3 ms
OJ-phase	It represents the reduction in the acceptor side of PS II
JI-phase	It represents the reduction in the PQ (Plastoquinone)
IP-phase	It represents the reduction in the acceptor side of PS I
Area	Area above the fluorescence induction curve
t_FM_	Time taken to reach F_M_
**Other JIP Parameters**	
F_V_ = F_M_ − F_0_	Maximal variable fluorescence (F_M_-F_O_)
F_V_/F_M_	It represents the maximum quantum yield of PSII
F_V_/F_O_	It represents the maximum efficiency of water-splitting complex
S_M_ = Area/(F_m_ − F_o_)	It represents the multiple turnovers of Q_A_ reductions
S_M_/t_FM_	It represents the average redox state of Q_A_ in the time span from 0 to t_FM_
N = Sm/ΔV/Δt_0_ *(1/VJ)	Turnover number of Q_A_ indicates the number of times Q_A_ was reduced from 0 to t_FM_
SFI _ABS_	An indicator of PS II structure and functioning
V_J_	Relative variable fluorescence at phase J of the fluorescence induction curve
V_I_	Relative variable fluorescence at phase I of the fluorescence induction curve
PI _ABS_ = γ_RC/_(1 − γ_RC_) * φ_Po_/(1 − φ_Po_) × ψ_o_/(1 − ψ_o_)	Performance index of PS II on absorption basis
PI _TOTAL_ = PI_ABS_ × δR_o_/(1 − δR_o_)	Performance index of electron flux to the final PS I electron acceptors
10RC/abs	Absorption per RC
RC/CS_M_	Density of active PS II reaction centres per cross-section
DF_ABS_ = log (PI_ABS_)	PSII-relative driving force index on an absorption basis
Kn	Non-photochemical de-excitation rate constant
Kp	Photochemical de-excitation rate constant
**Yield Parameters**	
φPo	Maximum quantum yield of primary PSII photochemistry (at t = 0)
φ (Do)	Quantum yield of energy dissipation
φ(Eo)	Quantum yield (at t = 0) for electron transport from QA^−^ to plastoquinone
ψo	Probability (at t = 0) that a trapped exciton moves an electron into the electron transport chain beyond QA
ϒRC	Probability that PSII chlorophyll molecule functions as RC
δ(Ro) = (1 − V_J_)/(1 − V_I_)	Efficiency/probability (at t = 0) with which an electron from the intersystem carriers moves to reduce end electron acceptors at the PSI acceptor side
**Specific Energy Flux**	
ABS/RC = (1 − γ_RC_)/γ_RC_	Absorption flux per RC corresponding directly to its apparent antenna size
TRo/RC = ΔV/Δt_0_ × (1/V_j_)	Trapping flux leading to QA reduction per RC at t = 0
ETo/RC = ΔV/Δt_0_ × (1/V_j_) ψ_0_	Electron transport flux from QA^−^ to plastoquinone per RC at t = 0
DIo/RC = (ABS/RC − TR_0_/RC)	Dissipated energy flux per RC at the initial moment of the measurement, i.e., at t = 0
**Phenomenological** **Energy Flux**	
ABS/CSm	Absorption of energy per excited cross-section (CS) approximated by F_M_
TRo/CSm	Excitation energy flux trapped by PSII of a photosynthesizing sample cross-section (CS) approximated by F_M_
ETo/CSm	Electron flux transported by PSII of a photosynthesizing sample cross-section (CS) approximated by F_M_
DIo/CSm	Heat dissipation of excitation energy by PSII of a photosynthesizing sample cross-section (CS) approximated by F_M_
